# Henry’s Law Constants and Vapor–Liquid Distribution Coefficients of Noncondensable Gases Dissolved in Carbon Dioxide

**DOI:** 10.1021/acsomega.1c07044

**Published:** 2022-03-02

**Authors:** Sergey B. Martynov, Richard T.J. Porter, Haroun Mahgerefteh

**Affiliations:** Department of Chemical Engineering, University College London, Torrington Place, London WC1E 7JE, U.K.

## Abstract

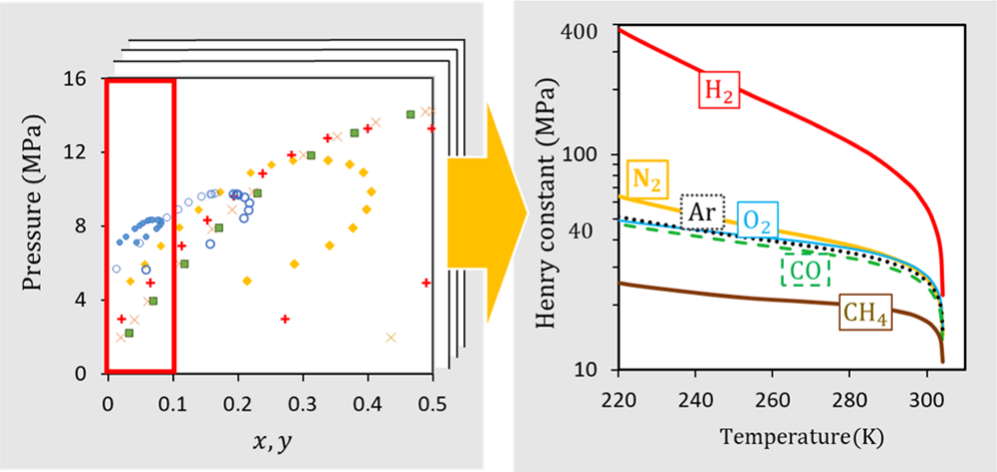

The accurate determination of the solubilities of the typical impurity gases present in captured CO_2_ in the carbon capture, utilization, and storage chain is an essential prerequisite for the successful modeling of the CO_2_ stream thermodynamic properties. In this paper, Henry’s law constants and the vapor–liquid distribution coefficients of six noncondensable gases, namely, N_2_, O_2_, H_2_, CH_4_, Ar, and CO, at infinite dilution in liquid CO_2_ are derived based on published vapor–liquid equilibrium data at temperatures ranging from the triple point (216.59 K) to the critical point (304.13 K) of CO_2_. The temperature dependence of Henry’s law constants of the six gases is correlated using approximating functions previously proposed for aqueous solutions. A correlation that provides the best fit for the Henry constants data for all the six gases, with the accuracy (absolute average deviation %) of 4.2%, is recommended. For N_2_, O_2_, H_2_, Ar, and CO, the combined standard uncertainty in the derived Henry constants is less than 6%, whereas for CH_4_, due to a larger deviation between the utilized data, the uncertainty is less than 18%. Analysis of the temperature variation of the vapor–liquid distribution coefficient at infinite dilution shows that when all the six gases are present in the CO_2_ stream, separation of N_2_, O_2_, Ar, and CO from CO_2_ can be problematic due to their similar volatilities, while the distinct volatilities of H_2_ and CH_4_ at lower temperatures make their separation from CO_2_ easier.

## 1 Introduction

Carbon capture, utilization, and storage (CCUS) refers to a collection of technologies proposed for reducing the CO_2_ emissions into the atmosphere from industrial installations and combustion-based power plants. Implementation of CCUS is recognized as an essential step in decarbonizing energy-intensive industries during the transition to renewable and other alternative energy sources. One of the key factors for the successful design of CCUS using process simulation tools is the availability of accurate thermodynamic property models for CO_2_ and its mixtures with other flue gas components. In particular, accurate modeling of the vapor–liquid phase equilibria (VLE) is critical for the appropriate design of the CO_2_ capture, purification, and compression processes^1−3^ as well as simulation of the flow behavior in the CO_2_ transportation and storage parts of the CCUS chain.^4^ Although models based on equations of state (EoSs) provide a reliable basis for predicting VLE data, their application, due to the complexity of the pertinent models and algorithms, can be difficult for use in engineering practice, for example, during the preliminary design of CO_2_ capture and purification processes or flow assurance calculations for pipeline transportation networks collecting CO_2_ streams of different purities from various industrial sources. In such cases, the calculation of phase equilibria in dilute solutions carrying small amounts of noncondensable gases, with dissolved gas mole fractions less than ca. 10%,^5^ can be performed with reasonable accuracy based on Henry’s law, as indeed widely used for the calculation of solubility of various components in aqueous solutions, for which extensive databases of Henry constants are readily available.^6^ However, with the exception of oxygen,^7,8^ xenon,^9^ and ozone,^10^ application of the above method to CO_2_ is hampered by the absence of experimental data on Henry’s law constants for the typical mixture components encountered in CCUS technologies.^11,12^ Obtaining the relevant experimental data is also important for validating theoretical estimates of the Henry constants, for example, those predicted based on the EoS or molecular dynamics models.^11,13^

The aim of the present study is to address the above knowledge gap by deriving Henry’s law constants from the VLE measurements for the typical to CCUS noncondensable gases mixed with CO_2_. At the infinite dilution limit, the Henry constants can be obtained from the slope of the bubble line in the *Px* diagrams^14^ or with the aid of the *Pxy* data—using the vapor–liquid ratios *y*/*x* extrapolated to the zero dilution limit.^15^ The latter approach is applied in the present study to determine Henry’s law constants for noncondensable gases, namely, nitrogen (N_2_), oxygen (O_2_), hydrogen (H_2_), methane (CH_4_), argon (Ar), and carbon monoxide (CO), which are typically found in CO_2_ streams captured from industrial installations, fossil fuel power plants, and oil refineries.^16^

Although the VLE data for binary mixtures of CO_2_ with the above-mentioned gases have been extensively studied, particularly for N_2_ and CH_4_, some of the reported data are inconsistent with each other^17^ and scant for CO_2_ mixtures with O_2_, Ar, H_2_, and CO.^18−20^ For binary mixtures of CO_2_ with NO and C_2_H_4_, the phase equilibria have not yet been experimentally characterized, although attempts have been made to predict the relevant VLE data using molecular dynamics.^21^

To obtain accurate and complete *Pxy* data covering a wide range of vapor and liquid mole fractions, several studies were performed in the past 15 years, resulting in the new measurements for CO_2_ binary mixtures with H_2_,^22^ N_2_,^23^ Ar,^24^ CH_4_,^25,26^ O_2_,^27^ and CO.^28−30^ In the present work, the results of these studies, particularly reporting the data on noncondensable components at small dilutions (below ca. 10% mol/mol), formed the basis for determining Henry’s law constants for gases dissolved in CO_2_.

In practice, the application of Henry’s law to binary solutions requires knowledge of the variation of the Henry constant with temperature and pressure. In particular, to describe Henry’s constant dependence on temperature at the solvent saturation pressure, two approaches are commonly applied.^31^ One of these is based on the integral form of van’t Hoff’s equation built around a specific temperature. Constructing this approximation requires knowledge of either the enthalpy of solution or the Henry constants at two points in the relevant temperature range. Alternatively, Henry’s constant variation over a wide range of temperatures is predicted using empirical correlations. For non-polar gases, relatively simple three-parameter semi-empirical approximations have been proposed to predict the Henry constant temperature dependence at the solvent saturation conditions, up to the solvent critical temperature.^13,32^ The effect of pressure on Henry’s law constants can be described using the Poynting correction, for example, in the form of the Krichevsky–Kasarnovsky equation.^33^ The above may become practically important when predicting the VLE at finite dilution. In the present study, approximations are constructed for the Henry constants over a wide range of temperatures to provide the basis for VLE calculations for noncondensable gases dissolved in CO_2_.

The rest of the paper is organized in three sections. Section 2 describes the method for obtaining Henry’s law constants and introduces correlations describing their variation with temperature. In Section 3, the relevant VLE data for binary mixtures of the six gases with CO_2_ are described and applied to obtain Henry’s law constants and the vapor–liquid distribution coefficient in the infinite dilution limit. For each solute, several correlations describing the temperature variation of Henry’s law constants are tested. Also, the uncertainties of newly obtained data and the accuracy of the proposed correlations are assessed. Section 4 presents the conclusions.

## 2 Methodology

### 2.1 Deriving Henry’s Law Constants from VLE Data

The solubility of a nonelectrolyte gas in a liquid in the limit of infinite dilution can be described by Henry’s law^5^
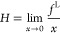
1where *f*^L^ and *x* are, respectively, the fugacity and the mole fraction of the solute in the liquid phase and *H* is the solute’s Henry’s law volatility constant, hereafter referred to as the Henry constant.

For a specific pair of solute and solvent, the Henry constant variation with pressure and temperature is described by the thermodynamic relations^34^
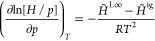
2
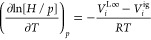
3where  is the heat of dissolution, represented by the difference of the partial molar enthalpies of a solute at infinite dilution and in the ideal gas state, *V*^L∞^ is the partial molar volume of a solute at the infinite dilution (where the subscript ∞ indicates the infinite dilution state), *V*^ig^ = *RT*/*p* is the partial molar volume of a solute in the ideal gas state, and *R* is the universal gas constant.

Using a suitable enthalpy of solution and partial molar volume data, the above equations can be integrated to fully characterize *H* as a function of pressure and temperature. In particular, knowing *H* at a reference pressure and specific temperature, integral forms of eq 3, for example, the Krichevsky–Kasarnovsky equation,^33^ can be used to obtain *H* values at elevated pressures.^34^ Integration of eq 2 can be performed to obtain the Henry constant variation with the temperature, for example, in the form of van’t Hoff’s law. However, since measurements of the heat of dissolution are usually not available, eq 2 is more frequently utilized for the opposite purpose, that is, characterizing the thermal effect of dissolution based on the Henry constant data. Such data are usually derived from *Pxy* measurements at various temperatures at infinite dilution, as described next.

At finite dilution, the liquid fugacity can be defined as^5^
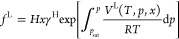
4where γ^H^ is the solute’s activity coefficient compatible with Henry’s law, *V*^L^ is the partial molar volume of the solute in the liquid phase, and *p*_sat_ is the saturation pressure of the pure solvent at a given temperature *T*.

Substituting eq 4 along with the definition of the vapor fugacity *f*^V^ = ϕ^V^*y p* into the fugacity balance equation *f*^V^ = *f*^L^ gives^5^
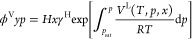
5where ϕ^V^ and *y* are, respectively, the solute fugacity coefficient and the mole fraction in the vapor phase. In the infinite dilution limit, *x* → 0, eq 5 reduces to
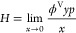
6

Based on this equation, with the aid of the *Pxy* data, *H* can be obtained by extrapolating isotherms  plotted as a function of *x* in the limit *x* → 0, as described by Wilhelm and Battino.^15^ This approach is adopted in the present study.

It can be noted that the ratio of the mole fractions in eq 6 is known as the distribution coefficient (also sometimes referred to as the partition coefficient, the absolute volatility, or *K*-value), *K* = *y*/*x*, which plays an important role in the VLE calculations for determining the composition and the bubble and dew points of a mixture and estimating the relative volatility of the components as a measure of their potential for separation in a distillation process. Noting that at infinite dilution the total pressure approaches the saturation pressure of pure solvent, *p*_sat_, *H* in eq 6 can be related to the distribution coefficient at infinite dilution, *K*^∞^
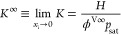
7where ϕ^V∞^ is the fugacity coefficient of the solute vapor phase at infinite dilution.

For each pair of solvent and solute, as in the case of the Henry constant, the infinite dilution distribution parameter *K*^∞^ can be approximated as a function of temperature. Remarkably, however, near the solvent critical point, *K*^∞^ correlates well with the solvent density^35,36^
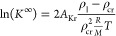
8where *A*_Kr_ is the Krichevskii parameter that has units of pressure, ρ_l_ and ρ_cr_ are, respectively, the saturated and critical densities of the solvent, and *M* is the molar mass of the solvent. Equation 8 shows implicitly the variation of *K*^∞^, and hence , with the temperature.

### 2.2 Temperature Dependence of the Henry Constant

Where the heat of dissolution remains approximately constant with temperature, the van’t Hoff eq 2 can be integrated to predict the Henry constant as a function of temperature. In practice, however, the heat of dissolution varies with temperature, and therefore, constructing such an approximation becomes useful only for capturing Henry’s constant variation around a specific temperature. To describe more accurately the Henry’s constant behavior over a wide range of temperatures, semi-empirical correlations have been proposed for non-electrolyte solutes in water and other solvents.^34,35^

In particular, Krause and Benson^37^ have suggested the three-parameter correlation for the Henry constant variation with temperature
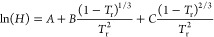
9where *A*, *B*, and *C* are the model parameters and *T*_r_ = *T*/*T*_cr_ is the reduced temperature based on the critical temperature of the solvent, *T*_cr_. At the solvent’s critical temperature, where the Henry constant takes the value *H*_cr_ ≡ *H*(*T*_cr_), eq 9 reduces to ln(*H*_cr_) = *A*, so that the constant *A* is determined by *H*_cr_. The latter can be calculated directly from eq 7 where the distribution coefficient *K*^∞^ turns to unity at the solvent critical point^38^
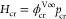
10where *p*_cr_ and  are, respectively, the solvent critical pressure and the corresponding vapor phase fugacity of the solute at infinite dilution.

Substituting the above expression for *H*_cr_ in eq 9 gives
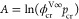
11

Based on the study by Krause and Benson^37^ and the results of a theoretical analysis of solubility of non-polar gases near a solvent’s critical point,^38,39^ Harvey^32^ has proposed approximating the Henry constant data in the following functional form
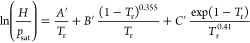
12where the first two terms on the right-hand side are aimed at capturing the temperature variation of *H* near the solvent critical temperature, while the third term is introduced as an empirical correction at low temperatures.

Trinh et al.^13^ have modified eq 12 to ensure that the last term vanishes in the limit *T* → *T*_cr_
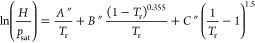
13

Advantageously, the constant *A*″ in the above equation is determined by an additional constraint set by the theoretical limit for *H*_cr_, defined by eq 10
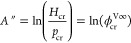
14

## 3 Results and Discussion

### 3.1 Deriving Henry Constants and Distribution Coefficients from Experimental Data

Following the approach described in Section 2.1, the Henry constant data were derived based on the *Pxy* measurements in binary mixtures of N_2_, O_2_, H_2_, CH_4_, Ar, and CO with CO_2_, obtained from primary literature sources. The pertinent experimental database was compiled based on most recent and most accurate measurements at small dilutions (*x* < 0.1)^5^ and temperatures between the triple point (216.59 K) to the critical point of CO_2_ (304.13 K). To ensure more complete coverage and better resolution of the temperature range, data from various sources were utilized, as, for example, in the case of the CO_2_–N_2_ mixture for which we used the data from Westman et al.^23^ and Fandiño et al.,^22^ the CO_2_–Ar mixture represented by the measurements from Løvseth et al.^24^ and Coquelet et al.,^43^ and the CO_2_–CO mixtures for which we combined the data from studies by Westman et al.,^28^ Chapoy et al.,^30^ and Souza et al.^29^ For CH_4_, the *Pxy* data from recent experimental campaigns were found to be rather limited. In particular, Legoix et al.^25^ have reported the bubble point data but did not include the dew point measurements, while the study by Peropoulou et al.^26^ has only covered temperatures above 293 K. To expand our database for CH_4_ to low temperatures, we have also included historical data identified in the recent literature reviews.^18,19^

For each solute gas, based on the *Pxy* data, the distribution coefficients *K* = *y*/*x* and the corresponding isotherms ϕ^V∞^*K p* were constructed and approximated using quadratic functions
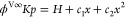
15where ϕ^V∞^ were approximated by the values calculated at very small dilutions (*x* = 0.0001) using the reference EoSs in REFPROP.^40^ The literature sources for the reference EoSs and the choice of mixing rules and specific parameters for each binary mixture are detailed in the Excel file in the Supporting Information.

**Figure 1:**
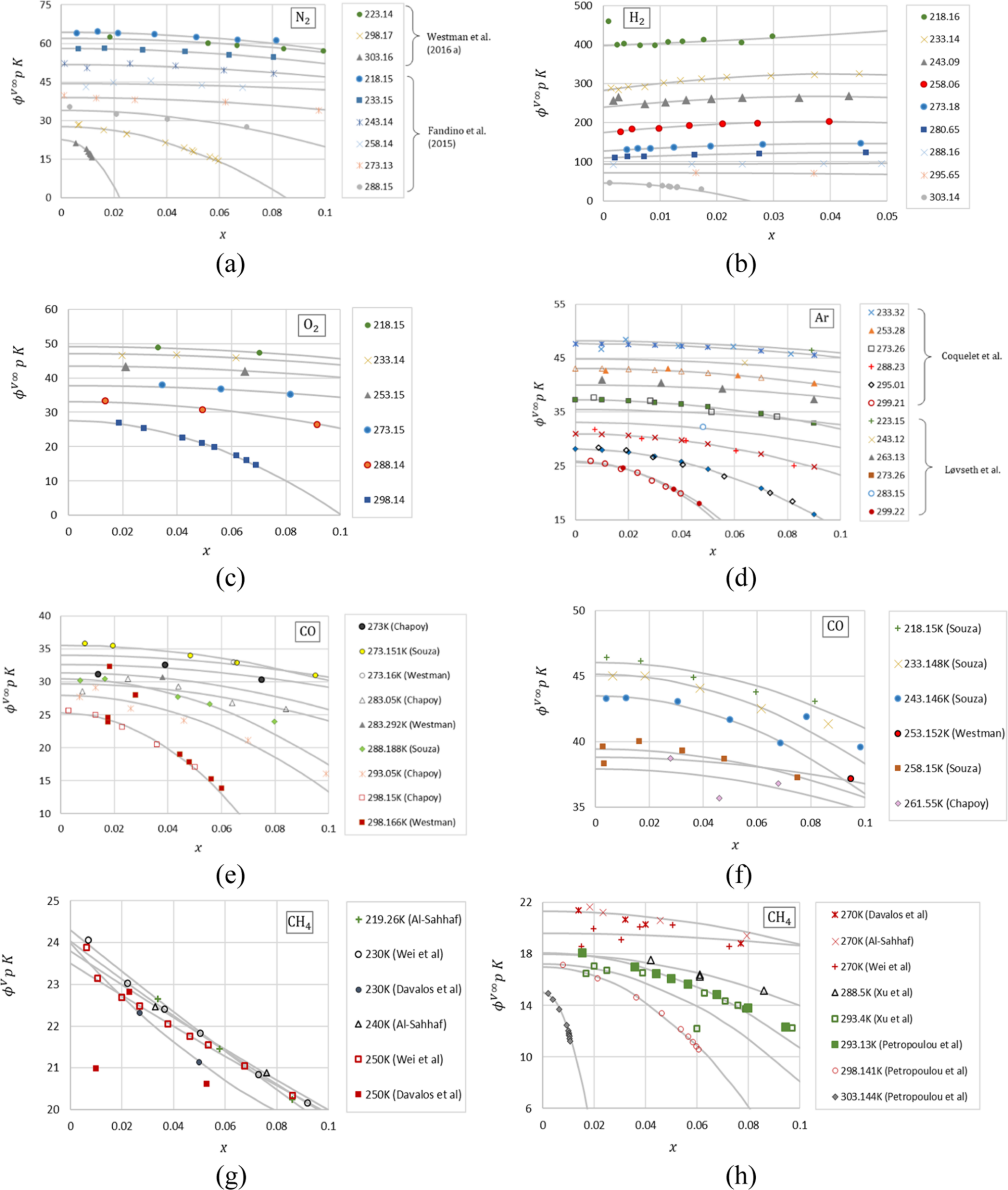
Variation of  as a function of the liquid mole fraction at different temperatures for different gases dissolved in CO_2_. (a) N_2_, (b) H_2_, (c) O_2_, (d) Ar, (e,f) CO, and (g,h) CH_4_. Points—experimental data derived from the *Pxy* measurements at various temperatures (see Table 1 for references), curves—fitted quadratic approximations using eq 15.

Figure 1 shows the ϕ^V∞^*K p* data plotted as a function of *x* and the corresponding approximations by eq 15 for various temperatures of the six gases, fitted using the least squares method in Excel (see the Supporting Information). Analysis of the data shows that although for all the gases sufficient data are available to resolve the isotherms at *x* < 0.1, some data at low *x* (particularly below 0.01) are scattered, deviating significantly from the trends observed at higher *x*. The points that deviated significantly from the data sets have been eliminated from further analysis. These included the measurements by Fandiño et al.^22^ for H_2_ at *x* = 0.0009, *T* = 218.16 K and *x* = 0.0026, *T* = 243.09 K (Figure 1b) and for CH_4_ by Davalos et al.^41^ at 230 and 250 K (Figure 1g) and Xu et al.^42^ at *x* = 0.0015, *T* = 288.5 K (Figure 1h).

As can be seen from the plots in Figure 1, eq 15 adequately approximates the data. The majority of the isotherms show parabolic behavior, with the exception of almost linear trends of some of the data for H_2_ (Figure 1b) and CH_4_ at low temperatures (Figure 1g). There is generally good consistency in the trends for isotherms obtained from different publications. Some of the data sets obtained by different authors at the same temperatures match with each other very well [e.g., the measurements by Coquelet et al.^43^ and Løvseth et al.^24^ for Ar (Figure 1d), by Souza et al.^29^ Westman et al.,^28^ and Chapoy et al.^30^ for CO, except for a small number of outliers in Westman et al.’s^28^ data at 253.152 and 298.166 K (Figure 1e,f), and by Pteropoulou et al.^26^ and Xu et al.^42^ for CH_4_ at 293 K (Figure 1h)]. The low dilution limit is particularly well resolved for H_2_ (Figure 1b) and also for N_2_ and Ar (Figure 1a,d, respectively). A relatively large scatter of the data can be observed in the measurements for CO (Figure 1e,f) and CH_4_ (Figure 1g,h).

Table 1 presents the list of *H* values fitted in eq 15 and the corresponding distribution coefficients, *K*_∞_, obtained for the six gases at different temperatures, along with the predicted values of the solutes’ fugacity coefficients and saturation pressures of CO_2_ calculated using eq 20 provided in the Appendix. The newly derived *H* and *K*_∞_ data are hereafter referred to as “experimental data”. Also included in Table 1 are the theoretical estimates for *H* obtained at the solvent critical point using eq 10 where *K*^∞^ turns to unity.^38^

**Table 1:** Values of the Henry Constants (*H*) and the Vapor–Liquid Distribution Coefficients at Infinite Dilution (*K*^∞^) Derived from the Literature *Pxy* Data for the Six Gas Solutes at Various Temperatures (*T*) and Calculated at the Critical Point of the CO_2_ Solvent Where  = 1 and *H*_cr_ Is Obtained from eq 10^a^

gas	reference	*T* (K)	*p*_sat_ (MPa)	*ϕ*^V∞^ (−)	*H* (MPa)	*K*^∞^ = (−)
H_2_	Fandiño et al. (2015)^22^	218.16	0.55	1.093	397.50	656.18
		233.14	1.00	1.139	282.94	247.38
		243.09	1.43	1.180	239.81	142.65
		258.06	2.28	1.264	175.01	60.60
		273.18	3.49	1.400	128.46	26.32
		280.65	4.23	1.504	109.69	17.25
		288.16	5.09	1.658	93.38	11.07
		295.65	6.07	1.929	72.43	6.18
		303.14	7.21	2.792	45.41	2.26
	theory @ *T*_cr_, eq 10	304.13	7.38	3.019	22.27	1
N_2_	Westman et al. (2016)^23^	223.14	0.68	1.004	62.05	90.59
		298.17	6.44	1.487	27.78	2.90
		303.16	7.21	1.863	22.72	1.69
	Fandiño et al. (2015)^22^	218.15	0.55	1.002	64.45	116.11
		233.15	1.00	1.011	57.96	57.05
		243.14	1.43	1.024	51.80	35.46
		258.14	2.29	1.058	44.49	18.37
		273.13	3.48	1.123	38.98	9.97
		288.15	5.09	1.260	33.89	5.29
		303.15	7.21	1.862	24.72	1.84
	theory @ *T*_cr_, eq 10	304.13	7.38	1.980	14.61	1
O_2_	Westman et al. (2016)^27^	218.15	0.55	1.051	49.09	84.37
		233.14	1.00	1.075	47.00	43.54
		253.15	1.97	1.126	43.39	19.57
		273.15	3.48	1.220	37.84	8.90
		288.14	5.09	1.369	33.07	4.75
		298.14	6.43	1.608	27.63	2.67
	theory @ *T*_cr_, eq 10	304.13	7.38	2.115	15.60	1
CH_4_	Petropoulou et al. (2018)^26^	303.14	7.21	1.411	15.04	1.48
		298.14	6.43	1.210	17.00	2.18
		293.13	5.73	1.137	18.07	2.78
	Xu et al. (1992)^42^	288.50	5.13	1.097	18.00	3.20
		293.40	5.76	1.139	17.22	2.62
	Davalos et al. (1976)^41^	270.00	3.20	1.006	21.32	6.47
	Al-Sahhaf et al. (1993)^44^	219.26	0.58	1.009	24.30	41.57
		240.00	1.28	1.028	23.80	18.44
		270.00	3.20	1.007	21.30	6.47
	Wei et al. (1995)^45^	230.00	0.89	1.007	24.04	26.76
		250.00	1.79	1.028	23.51	13.05
		270.00	3.20	1.006	19.61	5.95
	theory @ *T*_cr_, eq 10	304.13	7.38	1.476	10.89	1
Ar	Løvseth et al. (2018)^24^	223.15	0.68	1.037	48.20	68.15
		243.12	1.43	1.068	44.88	29.47
		263.13	2.65	1.125	40.06	13.45
		273.26	3.49	1.174	35.47	8.64
		283.15	4.50	1.249	33.06	5.88
		299.22	6.59	1.569	25.67	2.48
	Coquelet et al. (2008)^43^	233.32	1.01	1.050	47.71	44.94
		253.28	1.98	1.092	43.10	19.96
		273.26	3.50	1.174	37.29	9.09
		288.23	5.10	1.307	30.96	4.65
		295.01	5.98	1.431	28.14	3.29
		299.21	6.59	1.569	25.85	2.50
	theory @ *T*_cr_, eq 10	304.13	7.38	2.002	14.77	1
CO	Westman et al. (2018)^28^	253.15	1.97	1.106	38.84	17.82
		273.16	3.49	1.173	34.09	8.34
		283.29	4.52	1.237	31.38	5.62
		298.17	6.44	1.469	25.24	2.67
	Chapoy et al. (2020)^30^	261.55	2.53	1.128	37.91	13.28
		273.00	3.47	1.172	32.67	8.03
		283.05	4.49	1.235	29.76	5.37
		293.05	5.72	1.351	27.97	3.62
		298.15	6.43	1.468	25.34	2.68
	Souza et al. (2018)^29^	218.15	0.55	1.056	46.05	78.74
		233.15	1.00	1.072	45.14	41.90
		243.15	1.43	1.087	43.52	28.04
		258.15	2.29	1.119	39.43	15.39
		273.15	3.49	1.173	35.57	8.70
		288.19	5.09	1.284	30.50	4.67
		302.94	7.18	1.756	21.41	1.70
	theory @ *T*_cr_, eq 10	304.13	7.38	1.879	13.86	1

a *p*_sat_ is the saturation pressure of CO_2_, calculated using correlation by Span and Wagner provided in the Appendix.

### 3.2 Temperature Variation of the Henry Constants and the Distribution Coefficients

As can be seen in Table 1, both *H* and *K*^∞^ decrease with temperature. Given that *K*^∞^ and *H* are interrelated by eq 7, approximating the temperature variation for one of them is sufficient to determine the other. In this study, approximations for *H* are constructed using expressions of Section 2.2. Also, the validity of eq 8, as a basis for approximating *K*^∞^ and the corresponding *H* for a wide range of temperatures, is examined.

**Figure 2:**
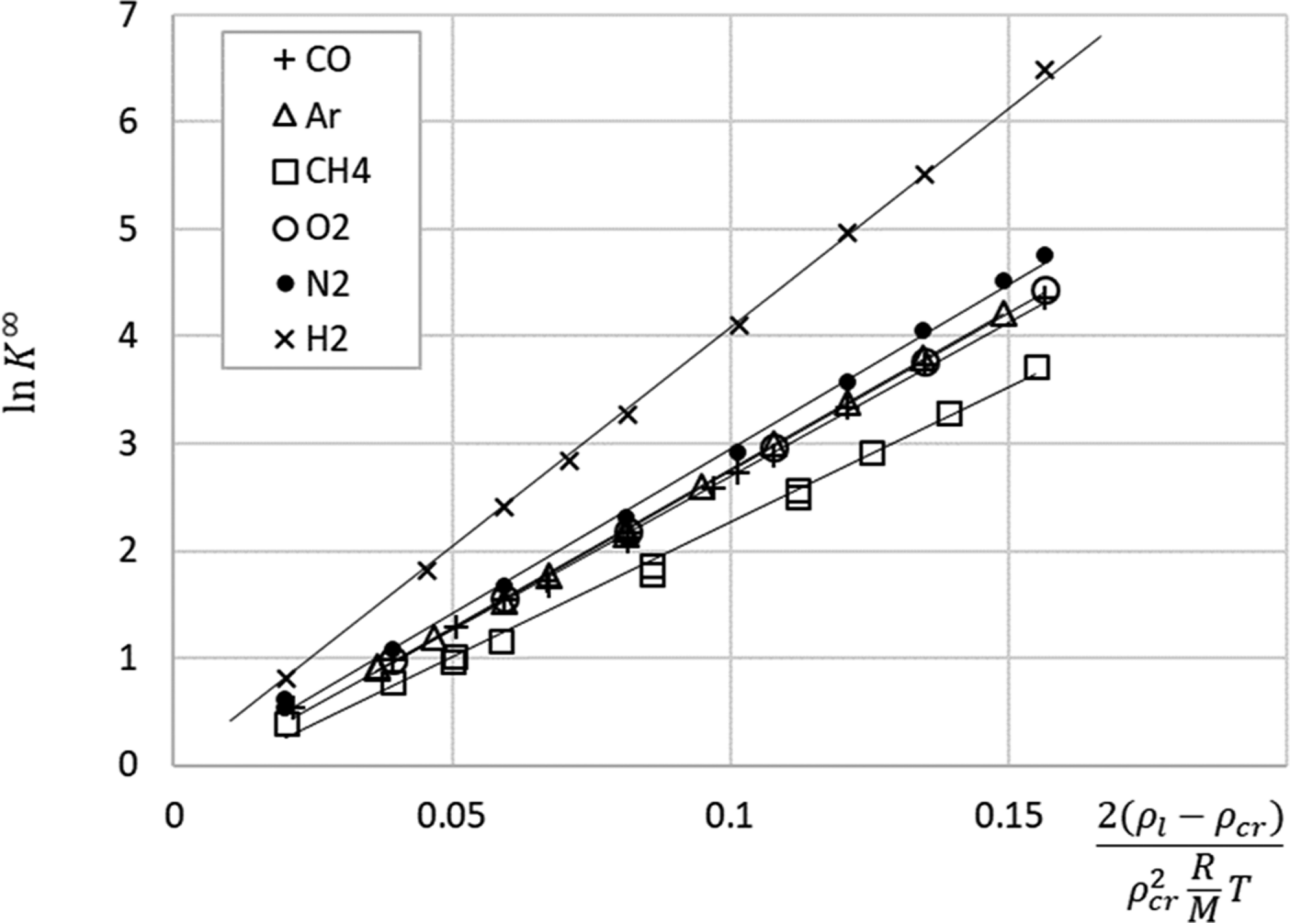
Variation of ln *K*^∞^ as a function of the dimensionless density of the solvent at saturation for the six gases. Points—data derived from the *Pxy* measurements obtained from various sources (Table 1), lines—fitted eq 8 with the coefficients provided in Table 2.

Figure 2 shows the variation of ln *K*^∞^ with  and the linear approximations constructed using the least squares regression of eq 8 for the six gases, performed using Excel (see the Supporting Information). The saturated liquid CO_2_ density was calculated using eq 21 presented in the Appendix. The fitted values of the Krichevskii parameter are listed in Table 2. Although Figure 2 gives an impression that the linear approximations fit the data well, it must be noted that relatively small approximation errors for ln *K*^∞^ can translate into relatively large uncertainties for *K*^∞^ and the corresponding Henry constant data, as discussed later in this section.

**Table 2:** Fitted Coefficients in eqs 8, 9, 12, and 13 Approximating the Henry Constant Variation with Temperature for Various Gases Dissolved in CO_2_ (Table 1)

gas	number of data points	Harvey’s correlation, eq 12			Trinh’s modification of Harvey’s correlation, eq 13			Krause and Benson’s (1989) correlation, eq 9			Equation 8
		*A*′	*B*′	*C*′	*A*″	*B*″	*C*″	*A*	*B*	*C*	*A*_Kr_ (MPa)
N_2_	10	–18.2	1.12	19.2	0.68	3.07	4.41	2.68	120	–111	29.69
H_2_	9	–19.2	2.14	20.6	1.11	4.54	4.01	3.10	84	316	40.86
O_2_	6	–12.2	1.90	13.0	0.75	2.87	3.64	2.75	123	–132	27.75
Ar	12	–13	1.78	13.8	0.69	2.88	4.12	2.69	114	–117	27.60
CO	16	–14.6	1.50	15.4	0.63	2.91	4.03	2.63	109	–113	26.90
CH_4_	12	–16.5	0.98	17.0	0.39	2.32	4.99	2.39	71	–83	22.82

The observed decrease in the *K*^∞^ values for the gases with the temperature (see Table 2) has important practical implications in the context of separating impurities from CO_2_ streams as part of the purification of captured CO_2_. In particular, the data in Table 1 and Figure 2 show that

- O_2_, CO, and Ar have very similar volatilities over the entire range of temperatures studied,
- the volatility of N_2_ is moderately (ca. 20%) higher than that of O_2_, CO, and Ar,
- the volatility of H_2_ is ca. 2–9 times higher than those of O_2_, CO, and Ar, and
- the volatility of CH_4_ is ca. 1.5–2.5 times lower than those of O_2_, CO, and Ar.

**Figure 3:**
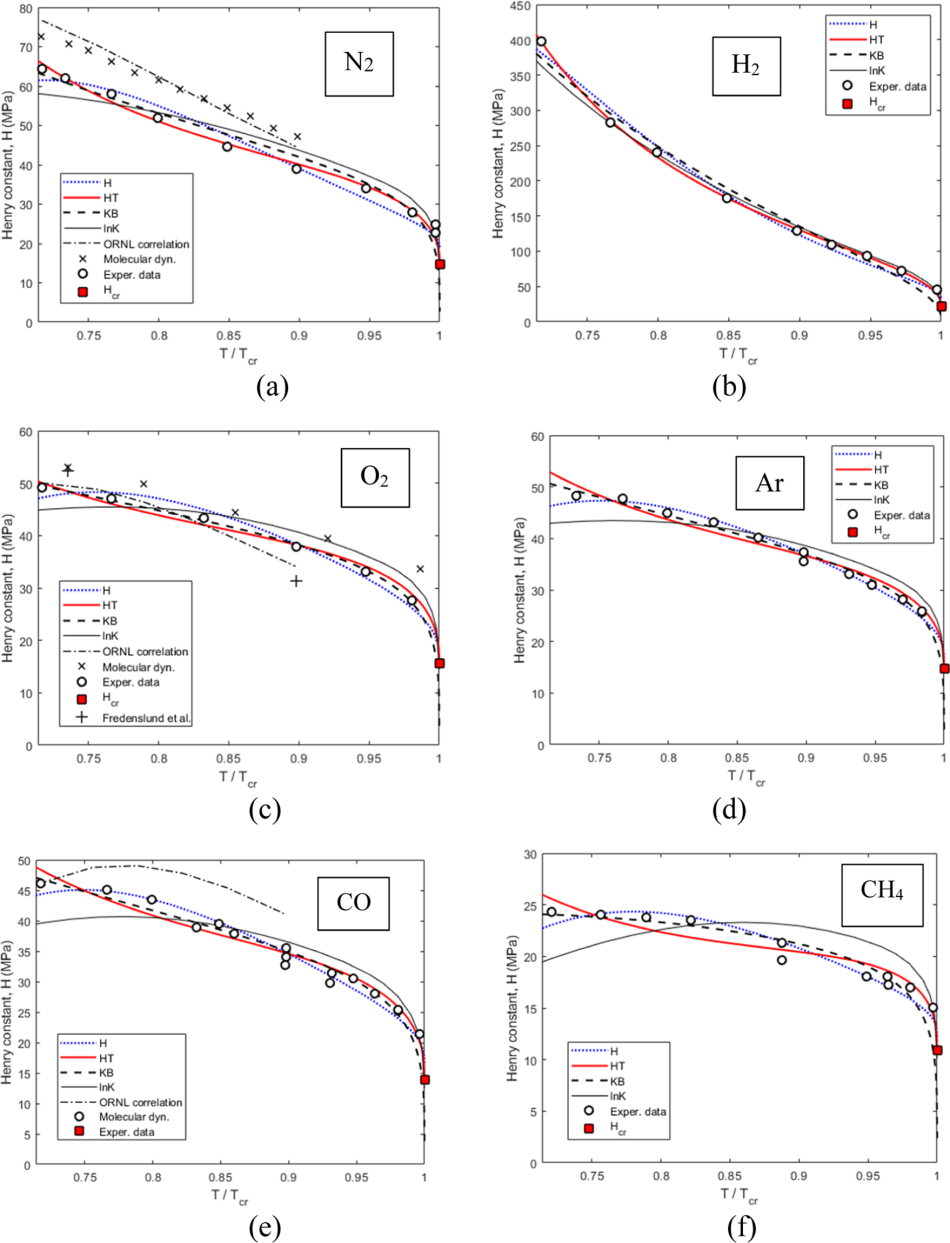
Henry constant variation with the temperature for various gases dissolved in CO_2_. (a) N_2_, (b) H_2_, (c) O_2_, (d) Ar, (e) CO, and (f) CH_4_. Data points from Table 1 (Exper. data) are presented along with the values reported for the O_2_–CO_2_ system at 273.15 K^8^ and 223.75 K,^7^ the molecular dynamics simulations for the N_2_–CO_2_ mixture^12^ and the O_2_–CO_2_ mixture,^11^ and the theoretical estimates of *H*_cr_ based on eq 10. Curves—fitted approximations: H (dotted blue curves)—Harvey’s^32^ correlation, eq 12, HT (solid red curves)—Trinh et al.’s^13^ modification of Harvey’s correlation, eq 13, KB (dashed black curves)—Krause and Benson’s^37^ correlation, eq 9, ln *K* (thin black curves)—eq 7 substituted in eq 8, ORNL correlation (black dashed–dotted curves) proposed by Glass and Barker^46^ (see the Supporting Information).

In Figure 3, the Henry constant values from Table 1, marked as “experimental data”, are plotted as a function of the reduced temperature of the solvent *T*_r_ = *T*/*T*_cr_, supplemented by the experimental data for O_2_,^7,8^ predictions based on the molecular dynamics simulations for N_2_^12^ and O_2_,^11^ the theoretical estimates of *H*_cr_ based on eq 10, and approximations by eqs 8, 9, 12, and 13 fitted to the “experimental data” using the least squares method in MatLab (see the Supporting Information). Table 2 presents the regressed coefficients in the approximating equations, while Table 3 presents the *R*^2^ statistics and the accuracy of the approximations expressed using the absolute average deviation (AAD).

**Table 3:** Statistics for the Constructed Approximations of the Henry Constant Variation with the Temperature (Table 2)^a^

gas	number of data points	Harvey’s correlation, eq 12		Trinh’s modification of Harvey’s correlation, eq 13		Krause and Benson’s (1989) correlation, eq 9		eqs 7 and 8		
		*R*^2^	AAD %	*R*^2^	AAD %	*R*^2^	AAD %	*R*^2^ (ln *K*)	AAD % (ln *K*)	AAD % (*H*)
N_2_	10	0.977	7.4	0.992	3.2	0.918	14.4	0.999	4.6	7.8
H_2_	9	0.992	9.9	0.999	2.3	0.983	19.1	0.9999	1.1	3.6
O_2_	6	0.986	3.6	0.992	2.3	0.803	12.7	0.999	4.1	7.2
Ar	11	0.990	2.9	0.983	3.1	0.869	8.4	0.999	4.5	7.5
CO	16	0.973	4.3	0.972	3.3	0.861	8.4	0.999	4.2	7.3
CH_4_	13	0.947	4.0	0.938	4.2	0.515	10.6	0.997	8.3	12.4

a , where  are the experimentally derived Henry constants (Table 1),  are the fitted data, and *N* is the number of points used to build the approximation.

As can be seen in Figure 3, eqs 9 and 13 both provide good approximations of the data in the entire temperature range investigated for all of the six gases. Equation 12 also approximates the data well but has the tendency of slightly underestimating the Henry constant values at *T*_r_ below ca. 0.75 (*T* < 230 K). The latter can likely be attributed to the fact that, as explained by Trinh et al.,^13^ the last term in Harvey’s eq 12 is not taken in the asymptotically correct form. In the case of oxygen (Figure 3c), the Henry constant data derived from the measurements by Westman et al.^27^ match well with the values reported by Fredenslund et al.^7,8^

Figure 3 also shows that correlations developed by the Oak Ridge National Laboratory (ORNL)^46^ for the Henry constant at low temperatures (below 273 K) provide a reasonably good approximation of the present data for O_2_ but overestimate by ca. 15% the data obtained in the present work for N_2_ and CO. It also can be seen that the molecular dynamics simulations^11,12^ tend to overestimate the Henry constants as compared to the present study, by ca. 10% for O_2_ and by ca. 20% for N_2_. These deviations are likely attributed to the accuracy of the experimental data used for tuning the molecular dynamics models.^11^

Returning to Table 3, the values of *R*^2^ appear to be close to unity for all the cases, indicating the adequacy of the form of adopted approximating functions. A comparison of AADs for the different approximations shows that on average, Trinh’s eq 13 gives the best fit over the entire range of temperatures (maximum AAD of 4.2%), followed by Harvey’s eq 12 (maximum AAD of 7.4%), and then eq 9 proposed by Krause and Benson (with the maximum AAD of 19.1%). In the case of approximation based on eq 8, the uncertainty in approximation of ln *K* almost doubles when translated to the linear scale, resulting in AAD % (*H*) in the range from 3.6 to 12.4% for the studied six gases.

Figure 3 shows that at *T*_r_ below ca. 0.75 (*T* < 230 K), eq 8 becomes less accurate than the other correlations and hence cannot be recommended for the calculation of Henry constants at temperatures far below the critical point of the solvent.

### 3.3 Uncertainty Analysis

The total standard uncertainty of the Henry constant data (*u*_tot_(*H*)) presented in Table 1 combines the propagated uncertainties of various quantitates that determine *H* in eq 6, *u*_exp_(*H*), and the uncertainty of fitting *H* values in eq 15, *u*_approx_(*H*)
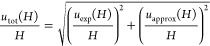
16

The first component, *u*_exp_(*H*), is estimated based on the analysis of the error propagation in eq 6
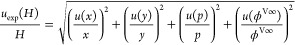
17where *u*(*x*), *u*(*y*), and *u*(*p*) are standard uncertainties of the corresponding experimentally observed quantities *x*, *y*, and *p* and *u*(ϕ^V∞^) is the uncertainty of ϕ^V∞^ estimated as described next.

Given that the fugacity coefficient is the quantity which is not measured directly but rather calculated based on the reference EoSs in REFPROP^40^ (see the details in the EOS validation section in the Excel file provided in the Supporting Information), the uncertainty *u*(ϕ^V∞^) carries the components associated with (a) the inaccuracy of the underlying predicting model and (b) the uncertainty propagated from the variables that the fugacity coefficient depends upon (e.g., pressure and composition). Given that the reference EoSs enable a very accurate representation of VLE data, at least at small dilutions (see the Supporting Information), it can be assumed that the corresponding uncertainty of predictions ϕ^V∞^ is negligibly small (although rigorous assessment of the accuracy of the fugacity coefficient model is beyond the scope of the present study) as compared to the experimental component of *u*(ϕ^V∞^) due to the propagation of the errors of the pressure measurements *u*(*p*)
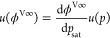
18

When using this equation, the derivative term was approximated as
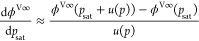
19

In eq 16, the second component of the combined total uncertainty, *u*_approx_(*H*), was obtained as the standard deviation of *H* fitted using the least square method in eq 15.

Table 4 presents the list of the individual components and the total combined uncertainty of *H* for the six gases at various temperatures. The relative uncertainty *u*_approx_(*H*)/*H* is generally smaller or of the same order of magnitude as the relative experimental uncertainty of the mole fraction measurements, while *u*(*p*)/*p* and *u*(ϕ^V∞^)/ϕ^V∞^ have the smallest contribution to *u*_tot_(*H*). For N_2_, O_2_, H_2_, Ar, and CO, the total combined uncertainty of the derived *H* values, *u*_tot_(*H*)/*H*, is less than ca. 4.7% (this applies to the majority of the data points, except for a few points near the critical temperature of CO_2_, where the uncertainty goes up to 6.13%), while for CH_4_, due to a larger relative uncertainty of the solute liquid fraction measurements, *u*(*x*)/*x*, the total combined uncertainty is the largest among the six gases studied (average 7%, maximum 18%).

**Table 4:** Relative Experimental Uncertainties of the Liquid and Vapor Mole Fractions [*u*(*y*)/*y* and *u*(*x*)/*x*], the Standard Uncertainty of Fitting the Henry Constants (*u*_approx_(*H*)/*H*) in eq 12, and the Total Standard Uncertainty of *H*, *u*_tot_(*H*), Estimated using eq 17^a^

gas	reference	*T* (K)	*H* (MPa)	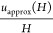 (%)	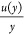 (%)	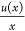 (%)	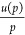 (%)	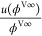 (%)	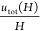 (%)
H_2_	Fandiño et al. (2015)^22^	218.16	397.50	1.00	0.67	2.08	0.54	0.02	2.47
		233.14	282.94	0.65	0.84	3.85	0.30	0.03	4.00
		243.09	239.81	1.28	0.88	2.94	0.21	0.03	3.33
		258.06	175.01	0.81	0.94	1.67	0.13	0.03	2.08
		273.18	128.46	0.51	1.00	1.22	0.09	0.04	1.66
		280.65	109.69	0.51	1.06	2.50	0.07	0.05	2.76
		288.16	93.38	0.17	1.08	2.94	0.06	0.07	3.14
		295.65	72.43	0.28	1.01	1.08	0.05	0.11	1.51
		303.14	45.41	1.77	2.00	4.55	0.04	0.25	5.30
N_2_	Westman et al. (2016)^23^	223.138	62.05	0.59	0.05	1.46	0.03	0.00	1.58
		298.174	27.78	1.02	1.64	4.21	0.02	0.02	4.64
		303.158	22.72	1.59	3.31	4.91	0.02	0.05	6.13
	Fandiño et al. (2015)^22^	218.147	64.45	0.23	0.68	1.09	0.31	0.00	1.34
		233.151	57.96	0.20	0.82	1.09	0.21	0.01	1.39
		243.138	51.80	1.09	1.05	3.85	0.20	0.01	4.14
		258.144	44.49	1.68	0.95	1.09	0.11	0.01	2.22
		273.128	38.98	0.71	1.09	4.55	0.08	0.02	4.73
		288.152	33.89	2.79	1.08	1.56	0.06	0.04	3.38
		303.153	24.72	2.68	1.09	2.00	0.04	0.13	3.53
O_2_	Westman et al. (2016)^27^	218.15	49.09	0.44	0.13	1.45	0.09	0.00	1.52
		233.14	47.00	0.32	0.12	2.48	0.05	0.00	2.50
		253.15	43.39	0.18	0.18	2.28	0.03	0.00	2.30
		273.15	37.84	0.82	0.64	1.39	0.03	0.01	1.74
		288.14	33.07	1.27	0.84	3.55	0.02	0.02	3.87
		298.14	27.63	0.56	1.10	2.58	0.02	0.03	2.86
CH_4_	Petropoulou et al. (2018)^26^	293.13	18.07	0.74	0.35	0.65	0.02	0.01	1.05
		298.141	17.00	0.65	0.70	1.24	0.02	0.01	1.57
		303.144	15.04	0.27	3.61	4.78	0.02	0.02	6.00
	Xu et al. (1992)^42^	288.5	18.00	1.64	1.92	6.67	0.75	0.26	7.17
		293.4	17.22	4.28	2.50	5.88	0.81	0.42	7.74
	Davalos et al. (1976)^41^	270	21.32	2.28	1.20	17.86	0.14	0.01	18.04
	Al-Sahhaf et al. (1993)^44^	219.26	24.30	0.76	0.57	5.90	0.14	0.00	5.98
		240	23.80	0.23	0.57	6.10	0.14	0.00	6.13
		270	21.30	0.93	1.91	10.93	0.14	0.01	11.13
	Wei et al. (1995)^45^	230	24.04	0.36	1.29	7.14	0.10	0.10	7.27
		250	23.51	0.20	2.56	7.69	0.10	0.10	8.11
		270	19.61	2.53	2.61	3.31	0.10	0.10	4.92
Ar	Løvseth et al. (2018)^24^	223.15	48.20	0.00	0.02	0.02	0.13	0.00	0.13
		243.12	44.88	1.30	0.02	0.24	0.02	0.00	1.32
		263.13	40.06	1.14	0.12	1.49	0.04	0.00	1.88
		273.26	35.47	0.00	0.04	0.12	0.02	0.00	0.13
		283.15	33.06	1.24	0.07	0.31	0.02	0.01	1.28
		299.22	25.67	0.63	0.36	0.83	0.02	0.03	1.10
	Coquelet et al. (2008)^43^	233.32	47.71	1.06	1.60	1.60	0.02	0.00	2.50
		253.28	43.10	0.60	1.60	1.60	0.01	0.00	2.34
		273.26	37.29	1.01	1.60	1.60	0.01	0.00	2.48
		288.23	30.96	1.38	1.60	1.60	0.01	0.00	2.65
		295.01	28.14	0.79	1.60	1.60	0.00	0.01	2.40
		299.21	25.85	0.48	1.60	1.60	0.00	0.01	2.31
CO	Westman et al. (2018)^28^	253.15	38.84	1.11	0.05	0.05	0.21	0.01	1.14
		273.16	34.09	1.24	0.10	0.50	0.03	0.01	1.34
		283.29	31.38	1.74	0.17	0.84	0.02	0.01	1.94
		298.17	25.24	0.74	0.66	1.81	0.02	0.02	2.07
	Chapoy et al. (2020)^30^	261.55	37.91	2.01	1.10	1.10	0.20	0.02	2.55
		273.00	32.67	1.37	1.10	1.10	0.20	0.04	2.08
		283.05	29.76	2.33	1.10	1.10	0.14	0.06	2.81
		293.05	27.97	2.32	1.10	1.10	0.14	0.11	2.80
		298.15	25.34	1.04	1.10	1.10	0.11	0.12	1.88
	Souza et al. (2018)^29^	218.15	46.05	0.68	1.10	1.10	1.62	0.04	2.35
		233.15	45.14	0.14	0.22	1.01	1.62	0.06	1.93
		243.15	43.52	0.29	0.19	0.90	1.62	0.08	1.89
		258.15	39.43	0.87	0.25	0.74	1.62	0.13	2.01
		273.15	35.57	0.59	0.42	0.54	1.62	0.31	1.88
		288.19	30.50	0.81	0.38	1.03	0.90	0.51	1.69
		302.94	21.41	1.33	0.31	0.83	0.90	1.80	5.29

a The relative standard uncertainties of the pressure measurements *u*(*p*)/*p* were obtained based on the experimental uncertainties *u*(*p*). The relative standard uncertainty of the fugacity coefficient *u*(ϕ^V∞^)/ϕ^V∞^ was calculated based on eq 18. The uncertainties *u*(*y*)/*y* and *u*(*x*)/*x* were either taken from the experimental studies^29,30,43^ or estimated as a ratio of the reported absolute experimental uncertainties divided by the smallest measured mole fraction. The uncertainty *u*(*x*) by Wei et al.^45^ was estimated to be 0.0005 based on the data reported to four significant figures.

## 4 Conclusions

The Henry constants and the infinite dilution vapor–liquid distribution coefficients were obtained for six noncondensable gas components, namely, N_2_, H_2_, O_2_, Ar, CO, and CH_4_, commonly present as impurities in the captured CO_2_ streams in the CCUS chain. The data were determined based on the most reliable *Pxy* measurements covering the entire range of practically relevant temperatures spanning from the triple point of CO_2_ (216.59 K) to its critical point (304.13 K) and the fugacity coefficients of the gases estimated using the reference EoSs in REFPROP.^40^

The accuracy of the derived Henry constants was found to be largely dependent on the quality of the utilized experimental *Pxy* data at small dilutions. The estimated combined uncertainty of the derived Henry constants is less than ca. 6% for N_2_, H_2_, O_2_, Ar, and CO. For CH_4_, the utilized experimental data carried relatively large uncertainties of the gas solubility measurements at small dilutions, resulting in the Henry constant uncertainties being as large as 18%.

The experimentally derived data were supplemented by the theoretical estimates for Henry constants at the critical temperature of the CO_2_ solvent (304.13 K). These estimates were found to be highly consistent with the experimentally derived data for all the gases studied, enabling better resolution of the temperature region near the critical point where the Henry constant attains its minimum.

Several empirical correlations, previously proposed for aqueous solutions, were tested to approximate the Henry constants’ variation with temperature. These included the correlations by Harvey,^32^ Krause and Benson,^37^ Trinh et al.,^13^ and the model based on the asymptotic behavior of the vapor–liquid distribution coefficient near the critical point utilizing the Krichevskii parameter. The correlation proposed by Trinh et al.,^13^ which is a modified version of Harvey’s^32^ equation, was found to best approximate the Henry constant variation with the temperature for all the six gases, with the estimated maximum AAD of 4.2%.

Along with the Henry constants, the vapor–liquid distribution coefficients of the gases in the limit of infinite dilution in CO_2_, which provide a measure of relative volatility of the solutes, were derived to aid the design of CO_2_ separation, purification, and transportation processes. Among the six gases examined, H_2_ has the highest volatility, followed by N_2_ and CO, Ar and O_2_ with very similar volatilities, and then CH_4_, which has the lowest volatility over the entire range of temperatures examined. The data obtained show that volatilities of all the gases decrease with the temperature. Remarkably, based on the results, it can be concluded that when all the six gases are present in the CO_2_ stream, separation of N_2_, O_2_, Ar, and CO from CO_2_ can be problematic due to their similar volatilities, while distinct volatilities of H_2_ and CH_4_ at lower temperatures make their separation from CO_2_ easier. This finding is important in the context of selecting the appropriate operating conditions and design of the gas stripping and purification steps for separation of noncondensable gases from the captured CO_2_ stream.

It is important to note that Henry’s law is a limiting law, and therefore, the Henry constants and the corresponding vapor–liquid distribution coefficients are defined in the limit of infinite dilution at the solvent saturation conditions. Based on Henry’s law, simple models can be constructed to enable the calculation of the VLE at finite dilutions. It should be noted that the accuracy of such models ultimately depends on the validity of the underlying assumptions and the closure models (predicting, e.g., the solute activity and fugacity coefficients) that need to be assessed by comparing the VLE predictions with the real data for a specific solute–solvent pair over the range of the gas solubilities of interest. Further work is needed to establish the ranges of validity of any Henry’s law-based models for the calculation of the VLE in CO_2_ solutions at finite dilutions.

Given the limited availability of the relevant *Pxy* data, the present study was limited to the characterization of the Henry constants only for the above listed six gases. For other noncondensable gases typically present in CO_2_ mixtures encountered in CCUS, such as, for example, NO and C_2_H_4_, the *Pxy* data are not available over a wide range of temperatures and need to be obtained, either experimentally or using verified molecular dynamics models,^11,21^ as a basis for the derivation of the Henry constants.

## Appendix

To calculate the saturation pressure of CO_2_, the equation proposed by Span and Wagner^47^ was applied

20

where *T*_cr_ = 304.1282 K and *p*_cr_ = 7.3773 MPa are the critical point temperature and pressure of pure CO_2_, while *a*_*i*_ and *n*_*i*_ are constants: *a* = −7.0602087, *a*_2_ = 1.9391218, *a*_3_ = −1.6463597, *a*_4_ = −3.2995634, *n*_1_ = 1, *n*_2_ = 1.5, *n*_3_ = 2, *n*_4_ = 4. This equation is valid in the range of temperatures from the triple point of CO_2_ (*T*_tr_ = 216.592 K) to the critical point, describing the saturation pressure with the relative uncertainty of 0.012%.

The density of saturated liquid CO_2_ was calculated as^47^

21

where ρ_cr_ = 467.6 kg/m^3^ is the critical density of pure CO_2_, while *a*_*i*_ and *n*_*i*_ are constants: *b*_1_ = 1.9245108, *b*_2_ = −0.62385555, *b*_3_ = −0.32731127, *b*_4_ = 0.39245142, *m*_1_ = 0.34, *m*_2_ = 1/2, *m*_3_ = 10/6, *m*_4_ = 11/6. This equation is valid in the range of temperatures from the triple point of CO_2_ to the critical point, describing the saturated density with the relative uncertainties of 0.015, 0.04, and 1% for temperatures below 295, 303 K, and the critical point, respectively.

## Supporting Information Available

Original and processed data (Martynov–Henry constants—supporting_information.xlsm) (XLS)
MatLab script for fitting the Henry constants as a function of temperature and plotting Figure 3 (Martynov–Henry constants—matlab_script.m) (TXT)
